# Dental Medicine

**Published:** 1892-01

**Authors:** R. M. Chase


### DENTAL MEDICINE.[1]

BY R. M. CHASE, D.D.S., M.D.

When invited by your committee to read a paper on dental medicine, I had hoped to find time to give, at least, a partial *résumé* of the many valuable drugs which the dental practitioner might call to his aid to alleviate human suffering, and by their means evoke a general discussion upon a subject which, to my mind, is sadly omitted in most dental conventions. While it is too large to be encompassed in one paper, or discussed in one session, my sole object is to speak of some of the later therapeutic agents which we, as specialists, may judiciously use to keep pace with others in general medicine and surgery.

Antiseptics I will place first on the list, as I believe you will bear me out when I make the assertion that those are first in importance to the dental practitioner, for without antisepsis no dentist can conscientiously practise his chosen profession. How to use, what to use, and when to use antiseptics in dentistry is a field large enough to occupy in discussion a whole session; hence I will give only a few of the large groups of agents which one may choose from, notably, carbolic acid, creosote, salicylic acid, eucalyptus oil, iodoform, bichloride of mercury, peroxide of hydrogen, benzoic acid, boracic acid, aristol, bromine, phenol sodique, campho-phenique, chloro-phenique, listerine, and Dr. Seiler's[2] antiseptic tablets, etc.

Nearly all of these, when brought into contact with disease-germs, destroy their vitality by arresting fermentative processes and preventing the decomposition of organic substances.

Hence it is our duty to select such as are more pleasant to use, antiseptic qualities being equal.

There are several elegant preparations in the market which, from their composition, are worthy of more than passing notice. Among these are listerine, campho- and bromo-phenique, hydrogen peroxide, etc. Listerine probably possesses a wider range of usefulness for the dentist than any one preparation, for, in fact, it may enter with propriety into nearly all prescriptions which the dentist may be called upon to write. As its composition is no secret, the practitioner is justified in prescribing it.

---

[1] Read before the New England Dental Society, October 29, 1891.

[2] *Medical Record*, February 18, 1888.

Tablets made from Dr. Seiler's formula are much handier, and I find them as effective, prescribed in proportion of one tablet to two ounces of water. This makes a convenient form of dispensing to the patient, and does away with bottles. For a mouth-wash, for ill-conditions of the oral cavities, it is unsurpassed. After extraction it prevents subsequent pain and soreness, and there is hardly a day passes that I do not prescribe it in some form. I prefer it on account of its agreeable qualities to phenol sodique, which was once a much-lauded and very good preparation, rightly used.

Campho-phenique, composed of fifty-one parts carbolic acid and forty-nine parts camphor gum, is a very elegant preparation for raw surfaces, pulp-canals, pyorrhœa alveolaris, and for exposed pulps before capping. Bromo-phenique I have used with good results in devitalized teeth when abscessed. The listerine, or antiseptic tablets, I use to clean my instruments, for the reason that it does not discolor as in the case with bichloride of mercury. If any of you present have not used listerine, I would recommend you to obtain literature upon it, which will give you a better idea than I can in a limited talk like this.

Peroxide of hydrogen still stands at the head as an antiseptic, and undoubtedly is one of the best agents yet discovered to annihilate germs.

Charles Marchand's preparation ($H_2O_2$) is, I believe, the best article in the market, as peroxide of hydrogen is very susceptible to certain conditions. To get good results, it should be kept in a cool place, well stoppered, and, when required for use, as much as desired should be poured from a large bottle into a small receptacle, and only what is to be used should be exposed to the light. When small cavities are to be cleansed, it should be injected with a small glass or rubber syringe, as metal should not be brought in contact with it, as it quickly destroys its utility. For reaching pulp-canals I find a small glass medicine-dropper very convenient, as by pressing upon the rubber bulb quickly it is forcibly ejected into the pulp-canal without much trouble. I used a wooden tooth-pick, reduced in size, to still further push it into the root. In treating all ill-conditions of the oral cavity, I make it a rule to first rinse thoroughly the mouth with dilute peroxide, and then apply remedies suitable for the same.

Aristol is a combination of iodine and thymol, manufactured by Friedrich Bayer & Co., Elberfeld, Germany. It is a valuable, inodorous, and non-toxic antiseptic remedy, much superior to iodoform, iodol, and sozo-iodol.

Aristol is insoluble in water and glycerin, slightly soluble in alcohol, readily so in ether and oil by the aid of friction. It is not absorbed into the economy, and is consequently non-toxic, in which respect it has the advantage over iodoform. It is a good germicide, and less irritating than most antiseptics. Its use in pulp-canals is especially indicated when there is considerable odor, as in case of a recently-decomposed pulp. It may be used either in powder or solution. The most important use I make of it is in combination with chloro-percha as a permanent root-filling, thus rendering the gutta-percha usually permanently antiseptic. Two grains of aristol powder incorporated into a drachm vial of the chloro-percha will suffice. If the aristol precipitates by standing, it should be well stirred together before using. While I do not claim that this is a specific for all trains of symptoms following the wake of devitalized or abscessed teeth, I do know that in my hands it comes nearest to an ideal root-filling of anything I have ever used. To illustrate: A lady came to me several months ago, anæmic and delicate, with a left superior second bicuspid, with the crown nearly all amalgam, with a vent hole near the gum on the buccal surface, which had been left open for several years. I advised her to have the root treated, filled, and a crown inserted. She looked up to me in surprise, and said, "I have been to several dentists, and they said they did not dare to stop up the root." I informed her that I was willing to undertake the operation, and did so. I proceeded to treat the tooth in the usual manner, after which I filled with aristol chloro-percha without the least irritation following.

I could cite many similar cases, also abscessed teeth, with like happy results.

Much more could be said, and undoubtedly will be brought out in this discussion, upon this and other valuable antiseptics. The other older antiseptics are too familiar to all to take up for discussion. Whatever is used, if it accomplishes asepsia, you have gained all that the best surgeons in the world can, and much may be added to our success in this direction as practitioners of dentistry.

Disinfectants and deodorizers are so closely allied to antiseptics that I will not arrange them separately, as most agents of these classes, with few exceptions, possess virtues in common with each other.

Before closing, you will pardon me if I refer briefly to a class of remedies which are held in high esteem by the general practitioner of medicine, and, while comparatively new, are fast superseding prescriptions of the past for the relief of many painful conditions which the dentist in his daily routine of practice is justified in treating.

Acetanilide (antifebrin) is a pure white and crystalline powder of neutral reaction, but of a slight burning taste. Acetanilide is a very efficient antipyretic, besides being strongly analgesic and antispasmodic. The two latter we, as dentists, are especially interested in. Its anodyne effect upon neuralgia of the fifth pair of nerves is often magical. For headache, after a protracted dental operation, it is especially useful. I usually give a patient a five-grain dose, either in a powder or in a more convenient form, compressed tablets, to be repeated every two hours until relieved. Not over thirty grains should be given in twenty-four hours. Usually one dose is sufficient for an ordinary headache. For a severe case of neuralgia several doses may be given, until the desired relief is obtained. I consider this superior, at the same time handier to prescribe, than gelsemium, aconite, etc., which have commonly been used for this troublesome affection.

Salol (salicylate of phenol), as its name implies, is of the phenol family, and is another valuable remedy for neuralgia, consisting of sixty parts by weight of salicylic acid and forty of carbolic acid, is insoluble in water, almost tasteless, and odorless. Dose, five to ten grains, once in two hours, until three or four doses have been taken.

Antikamnia, meaning "opposed to pain," is a new analgesic, antipyretic and anodyne, furnished by the Antikamnia Chemical Company of St. Louis, and is valuable in neuralgia and other allied disorders. It relieves pain and has no after-effect, causing no excitation of the heart or cyanosis. Dose, three to ten grains, followed by a swallow of water. This remedy may be used in all cases as a substitute for morphia, without the after-effects of opiates. It can safely be exhibited, being given in from twenty-four to sixty grains during twenty-four hours.

Sulphonal is a very popular, prompt, safe, and reliable hypnotic, which, in proper doses, produces quite natural sleep. This valuable remedy may be best given when patients suffer from persistent neuralgia, in doses from ten to thirty grains. This remedy has not the ill effects of morphine, and the dose need not be increased by long-continued use. I believe that it is one of the best hypnotics that we possess. Sulphonal is best administered at supper-time, dissolved in hot liquids; usually a cup of tea makes a good vehicle. It may be obtained in tablets of five and fifteen grains, which make a very convenient way of prescribing. It is unquestionably the best article that we have to prescribe to a nervous patient after a protracted operation in the chair, as a small dose will give them a quiet, peaceful night's rest.

Phenacetin is another valuable analgesic which the dental practitioner may call to his aid. I have had good results in administering it in five-grain doses, once in one hour, until fifteen to twenty grains have been given. This seems to control neuralgia and headache when other remedies fail. While some administer it as an analgesic in single doses of fifteen or twenty grains, I have not had occasion to use such large doses to control my cases. It is absolutely tasteless, and more agreeable to take than any other antipyretic, and without the toxic effects which are claimed of some of the other preparations of this class.

With these few remarks I will close, trusting that the remedies which I have noted will, at least, serve to start a discussion which will undoubtedly bring out many other articles in *materia medica*, which will be of inestimable worth to us as practitioners of dental surgery.

